# Lung Imaging Data Analysis

**DOI:** 10.1155/2013/618561

**Published:** 2013-02-28

**Authors:** Ayman El-Baz, Garth M. Beache, Georgy Gimel'farb, Kenji Suzuki, Kazunori Okada

**Affiliations:** ^1^BioImaging Laboratory, Bioengineering Department, Speed School of Engineering, The University of Louisville, Louisville, KY 40292, USA; ^2^Department of Radiology, School of Medicine, The University of Louisville, Louisville, KY 40292, USA; ^3^Department of Computer Science, The University of Auckland, Auckland 1142, New Zealand; ^4^Department of Radiology, Division of Biological Sciences, The University of Chicago, Chicago, IL 60637, USA; ^5^Department of Computer Science, San Francisco State University, San Francisco, CA 94132, USA

Early detection and diagnosis of lung cancer, causing most of cancer-related deaths worldwide, can improve effectiveness of the treatment and increase chances of patients to survive. Comparing to conventional chest radiography, advanced lung imaging modalities, for example, low-dose helical CT, cone-beam CT, and PET (positron emission tomography), together with the progress of image analysis techniques provide the means for detecting smaller pulmonary nodules and at earlier stages. These developments help in diagnosing other diseases and conditions, such as chronic obstructive lung disease, interstitial lung disease, and pulmonary embolism, too.

Seven papers selected for this special issue cover different aspects of early lung cancer detection and diagnostics, such as registration and segmentation of lung regions and motion correction, detection, and diagnosis of lung nodules. The paper “*Computer-aided diagnosis systems for lung cancer: Challenges and methodologies*” by A. El-Baz et al. reviews the current state-of-the-art data processing techniques at each CAD stage and outlines, for each technique, methodologies and technical issues of implementation, training and testing databases, validation methods, and achieved performances. Some implementation challenges and strengths and drawbacks of the known lung cancer CAD systems are also discussed.

O. J. Ilegbusi et al. present in their paper “*Modeling airflow using subject-specific 4DCT-based deformable volumetric lung models*” a subject-specific modeling of lung tumors and adjacent tissues motions based on computational fluid dynamics (CFD). Both the airflow and the associated structural deformation of the lungs were described simultaneously with an airflow-tissue interaction model. The lung was modeled as an anisotropic poroelastic medium and its geometry at the end-expiration was reconstructed from a 4DCT dataset of patients with non-small-cell lung cancer (NSCLC). Their results confirmed capabilities of the proposed model to fully represent and quantify detailed motion at any location inside the lungs.

The paper “*Improving intensity-based lung CT registration accuracy utilizing vascular information*” by K. Cao et al. correlates fluid redistribution and reabsorption to changes in regional lung function by image matching. Lung CT datasets from six human subjects, scanned at four time instants before and after the bronchoalveolar lavage (BAL) process, have been used. Dense displacement fields obtained from aligning images at the different instants and for various inflation levels via interphase and intraphase image registration are then used to track tissue volume changes and quantify the deformation patterns of local parenchymal tissue. 

The paper “*Quantification of lung damage in an elastase-induced mouse model of emphysema*” by A. Muñoz-Barrutia et al. presents a framework that defines the sensitivity of descriptors derived from micro-CT for quantifying lung damage caused by elastase instillation. Test samples for the proposed framework were analyzed using breath-hold-gated micro-CT, pulmonary function tests (PFTs), RT-PCR for RNA cytokine expression, and histomorphometry. The framework provides software for automatic quantification of airspace enlargement in large histological tissue sections.

A nonrigid pulmonary image registration algorithm proposed by K. Cao et al. in the paper “*Improving intensity-based lung CT registration accuracy utilizing vascular information*” exploits common intensity-based lung registration criteria, a feature-based constraint on vessels and a linear elastic smoothing constraint. The vessel-related constraint is integrated into the registration to correct mismatches of small lung vessels and their surrounding tissues. The algorithm is useful for tracking the lung motion between a pair of intrasubject CT images acquired at different inflation levels.

The paper “*Automated lobe-based airway labeling*” by S. Gu et al. presents an automatic classification of airways into five pulmonary-lobe-related categories. Data processing stages include airway skeletonization, individual airway branch identification, initial rule-based airway classification (labeling), and self-correction of the labeling errors. Their approach was tested on 300 chest CTs, and an image analyst examined the results. The labeling accuracy was 100% for the right upper lobe, 99.3% for the right middle lobe, 99.3% for the right lower lobe, 100% for the left upper lobe, and 100% for the left lower lobe. One airway tree was labeled in 2 min.

An algorithm for detecting lung nodules in chest CT scans presented in the paper “*Automatic detection of 2D and 3D lung nodules in chest spiral CT scans*” by A. El-Baz et al. starts by isolating the lung nodules, arteries, veins, bronchi, and bronchioles from the surrounding anatomical structures. Then the lung nodules are detected using deformable 3D and 2D templates describing typical geometry of and a typical gray-level distribution within the nodules. Finally, the false positive nodules are eliminated using three features that robustly define the true lung nodules.

While these papers in no way exhaust the broad and challenging area of early detection and diagnosis of lung cancer, they present rich and many-faceted knowledge that beyond any doubt deserves close attention of the readers. 

